# Effect of Substituents of Cerium Pyrazolates and Pyrrolates on Carbon Dioxide Activation

**DOI:** 10.3390/molecules26071957

**Published:** 2021-03-31

**Authors:** Uwe Bayer, Adrian Jenner, Jonas Riedmaier, Cäcilia Maichle-Mössmer, Reiner Anwander

**Affiliations:** Institute of Inorganic Chemistry, Eberhard Karls Universität Tübingen, Auf der Morgenstelle 18, 72076 Tübingen, Germany; uwe.bayer@uni-tuebingen.de (U.B.); adrian.jenner@student.uni-tuebingen.de (A.J.); jonas.riedmaier@student.uni-tuebingen.de (J.R.); caecilia.maichle-moessmer@uni-tuebingen.de (C.M.-M.)

**Keywords:** cerium, pyrazoles, pyrroles, carbazoles, carbon dioxide

## Abstract

Homoleptic ceric pyrazolates (pz) Ce(RR’pz)_4_ (R = R’ = *t*Bu; R = R’ = Ph; R = *t*Bu, R’ = Me) were synthesized by the protonolysis reaction of Ce[N(SiHMe_2_)_2_]_4_ with the corresponding pyrazole derivative. The resulting complexes were investigated in their reactivity toward CO_2_, revealing a significant influence of the bulkiness of the substituents on the pyrazolato ligands. The efficiency of the CO_2_ insertion was found to increase in the order of *t*Bu_2_pz < Ph_2_pz < *t*BuMepz < Me_2_pz. For comparison, the pyrrole-based ate complexes [Ce_2_(pyr)_6_(*µ*-pyr)_2_(thf)_2_][Li(thf)_4_]_2_ (pyr = pyrrolato) and [Ce(cbz)_4_(thf)_2_][Li(thf)_4_] (cbz = carbazolato) were obtained via protonolysis of the cerous ate complex Ce[N(SiHMe_2_)_2_]_4_Li(thf) with pyrrole and carbazole, respectively. Treatment of the pyrrolate/carbazolate complexes with CO_2_ seemed promising, but any reversibility could not be observed.

## 1. Introduction

Rare-earth–metal complexes are capable of efficiently activating carbonylic compounds including carbon dioxide [1,2,3,4,5,6]. Due to its environmental impact, atmospheric CO_2_ management and, in particular, sustainable solutions for CO_2_ emission control evolved as a top-priority issue in academic and industrial research [7,8,9,10,11]. On the one hand, this can be realized by capturing and storing carbon dioxide with tailor-made surface-reactive materials [12,13,14,15,16]. On the other hand, the use of CO_2_ as a cheap, abundant, and nontoxic C1 building block in the synthesis of higher-value chemicals is a main goal in sustainable chemistry [17,18,19,20,21,22,23]. For example, rare-earth metals have successfully been studied as catalysts for the copolymerization of carbon dioxide and epoxides to yield polycarbonates [24,25,26,27,28,29,30]. Various rare-earth-metal-based (pre)catalysts are known to promote the catalytic cycloaddition of carbon dioxide and epoxide-producing cyclic carbonates, which then, in case of propylene carbonate, can be used as electrolyte solvent in lithium-ion batteries [30,31,32,33,34,35,36,37]. However, these complexes often lack the catalytic activity and superb performance of zinc or cobalt-based systems [4].

**Figure 1:**
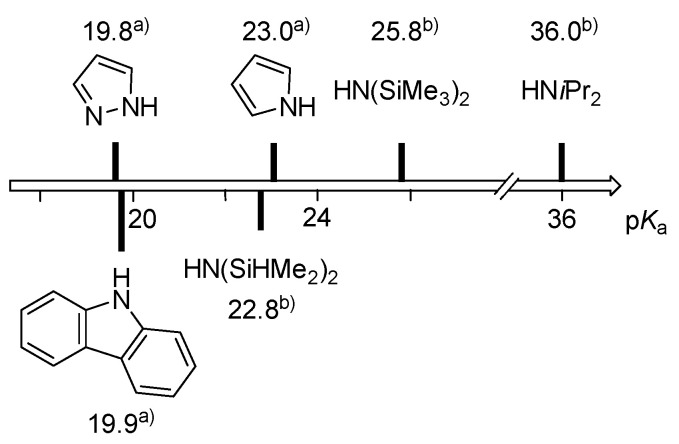
p*K*_a_ values of the different *N*-proligands [40,63,64]. ^a)^ Determined in DMSO, ^b)^ determined in THF.

We have recently described the application of homoleptic ceric pyrazolate [Ce(Me_2_pz)_4_]_2_ in the catalytic cycloaddition of carbon dioxide and epoxides as well as the reversible capture of CO_2_ [38,39]. To study the scope and efficiency of such pyrazolate-promoted CO_2_ insertion reactions, we extended our study with a broader comparison to differently substituted pyrazole derivatives as ligands for cerium(IV). Since we hypothesized that the basicity of the ligand plays a crucial role in any reversible CO_2_ uptake, we also envisaged different *N*-proligands. Pyrroles feature a mono-aza five-membered ring, exhibiting a p*K*_a_ value that is larger than that of pyrazoles, but close to silylamines (Figure 1) [40]. Metal silylamides, however, were shown to engage in a cascade of reactions with CO_2_, ultimately affording metal siloxides [41,42,43]. Whereas pyrrole-derived pincer-type ligands are quite popular in rare-earth–metal coordination chemistry [44,45,46,47,48,49,50,51], examples of pure pyrrolyl ligands have remained scarce [52,53,54,55]. Likewise, rare-earth–metal complexes bearing a carbazolyl ligand have been reported [56,57,58,59,60,61,62]. As a pyrrole derivative, carbazole exhibits a p*K*_a_ value that matches that of pyrazole (Figure 1).

## 2. Results and Discussion

### 2.1. Homoleptic Ceric Pyrazolates

**Scheme 1:**
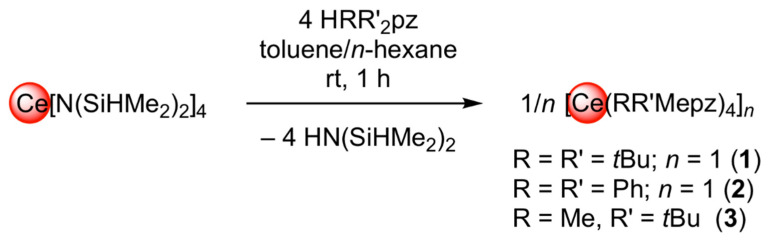
Synthesis of ceric pyrazolates **1** to **3**.

Homoleptic cerium di-*tert*-butyl pyrazolate Ce(*t*Bu_2_pz)_4_ (**1**) was synthesized by treatment of Ce[N(SiHMe_2_)_2_]_4_ with four equivalents of *t*Bu_2_pzH as previously described (Scheme 1) [65]. Likewise, Ce(Ph_2_pz)_4_ (**2**) and Ce(*t*BuMepz)_4_ (**3**) were synthesized using the corresponding pyrazole (Scheme 1). The ^1^H NMR spectrum of **2** shows a singlet for the proton in the pyrazolato backbone at 7.11 ppm and two multiplets at 6.91 and 7.78 ppm for the aromatic protons (Figure S1, Supporting Information). In contrast to the complexes bearing symmetric pyrazolato ligands, compound **3** with the asymmetric 3-*tert*-butyl-5-methyl pyrazolato ligand could not be obtained as a crystalline material, but as a dark red sticky solid upon removing the volatiles in vacuo. The general composition of [Ce(*t*BuMepz)_4_]*_n_* was confirmed by ^1^H NMR spectroscopy showing singlets at 1.24 ppm for the *t*Bu groups, at 2.22 ppm for the methyl groups, and at 6.15 ppm for the C−H proton of the five-membered pyrazole ring (Figure S2, Supporting Information). However, elemental analysis displayed some extent of impurification, indicated by an increased carbon value most likely stemming from retained solvent.

**Figure 2:**
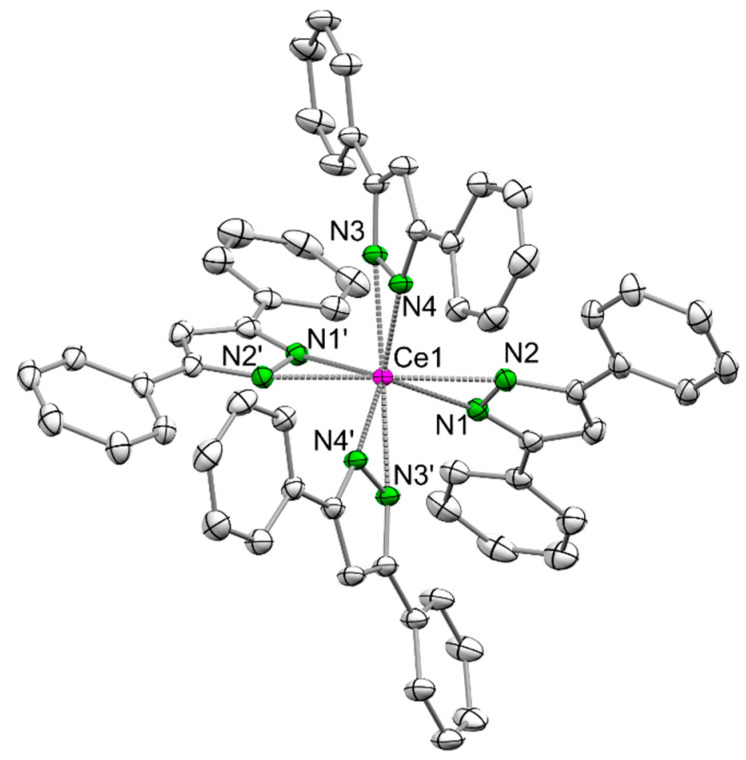
Crystal structure of Ce(Ph_2_pz)_4_ (**2**). Ellipsoids are shown at the 50% probability level. Hydrogen atoms and lattice toluene are omitted for clarity. Selected interatomic distances (Å) and angles (°): Ce1−N1 2.3790(16), Ce1−N2 2.3381(16), Ce1−N3 2.3409(15), Ce1−N4 2.3575(16); N1−Ce1−N3 113.41(6), N1−Ce1−N4 118.73(5), N2−Ce1−N3 96.05(6), and N2−Ce1−N4 87.76(5).

Crystals of complex **2** suitable for an X-ray diffraction (XRD) study could be obtained from a concentrated solution in toluene. The crystal structure revealed an eight-coordinate cerium center surrounded by four *η*^2^-coordinated diphenylpyrazolato moieties (Figure 2). The Ce1–N distances (2.3381(16) to 2.3790(16) Å) are comparable to other terminal Ce^IV^–N(pz) bonds (**1**: 2.322(4) to 2.365(4) Å; Ce(Me_2_pz)_4_: 2.319(3) to 2.384(2) Å) [65].

### 2.2. Cerium Pyrrolates and Carbazolates

**Scheme 2:**
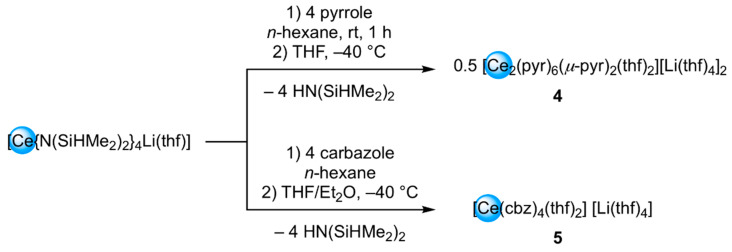
Synthesis of cerous pyrrolate and carbazolate ate complexes **4** and **5**.

As the synthesis pathway via protonolysis reaction of Ce[N(SiHMe_2_)_2_]_4_ with pyrazole emerged as a feasible route for the synthesis of homoleptic ceric pyrazolates, the envisaged pyrrolates were accessed accordingly. Unexpectedly, treatment of the silylamide with four equivalents of pyrrole did not yield any reaction. Therefore, we reacted the cerous ate complex Ce[N(SiHMe_2_)_2_]_4_Li(thf) [66] with four equivalents of pyrrole (Hpyr) and carbazole (Hcbz), yielding the complexes [Ce_2_(pyr)_6_(*µ*-pyr)_2_(thf)_2_][Li(thf)_4_]_2_ (**4**) and [Ce(cbz)_4_(thf)_2_][Li(thf)_4_] (**5**), respectively (Scheme 2). The ^1^H NMR spectrum of **4** shows two broadened singlets for the pyrrolato protons at 4.23 and 7.39 ppm (Figure S3, Supporting Information). For complex **5**, various broadened proton signals could be detected as well but due to the paramagnetic Ce^III^ center, assignment of the signals was inconclusive (Figure S5, Supporting Information). The persistence of ion-separated intermolecular ate complexes in THF-*d*_8_ was evidenced by ^7^Li NMR spectroscopy (**4**: δ_Li_ = 2.0 ppm; **5**: δ_Li_ = −0.3 ppm; (Figures S4 and S6, Supporting Information)) [67].

**Figure 3:**
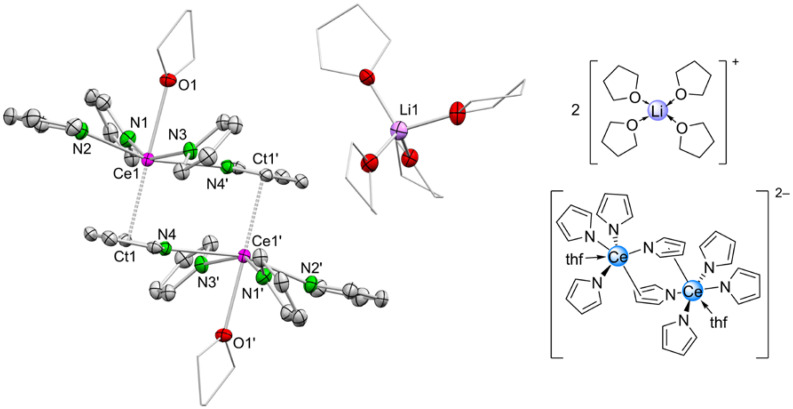
Crystal structure of [Ce_2_(pyr)_6_(*µ*-pyr)_2_(thf)_2_][Li(thf)_4_]_2_ (**4**). Ellipsoids are shown at the 50% probability level. Hydrogen atoms and the second [Li(thf)_4_]^+^ counter ion are omitted for clarity. Selected interatomic distances (Å) and angles (°): Ce1−N1 2.512(2), Ce1−N2 2.475(2), Ce1−N3 2.515(2), Ce1−N4′ 2.622(2), Ce1−Ct1 2.575(16), Ce1−O1 2.5138(17); N1−Ce1−N2 97.21(7), N1−Ce1-N3 153.53(8), N1−Ce1−N4 86.15(7), N1−Ce1−O1 78.80(7), and N4−Ce1′−Ct1′ 97.8(5).

Crystallization from a concentrated THF solution gave **4** as colorless crystals. The crystal structure of **4** revealed a separated ion pair featuring a dicerium dianionic entity (Figure 3). Each cerium center is eight-coordinated and surrounded by three terminal pyrrolato moieties (Ce1–N 2.475(2) to 2.515(2) Å), two bridging pyrrolato ligands connecting the cerium centers in an *η*^1^ (Ce1–N4′ 2.622(2) Å) and ^η5^ fashion (Ce1–Ct 2.575(16) Å), and one THF donor molecule. The Ce–N distances are slightly elongated compared with other Ce^III^ ate complexes like [Ce{N(SiHMe_2_)_2_}_4_][Li(do)*_x_*] (do, *x* = py, 4; tmeda, 2; 12-crown-4, 1 and thf, 4; Ce–N 2.377(6) to 2.438(6) Å), KCe[N(SiHMe_2_)_2_]_4_ (2.3820(12) to 2.4379(12) Å), and [Ce{N(SiMe_3_)_2_}_4_][Na(thf)_4_(Et_2_O)] (Ce–N 2.434(6) to 2.448(6) Å), due to the higher coordination number CN of **4** (CN 8 vs. 4) [67,68,69]. The σ,π-bridging motif of the pyrrolato ligands has been detected previously, for example, in complexes [Me_2_In(*µ*-pyr)]*_n_*, [Me_2_Al_2_(*µ*-Cl)](*µ*-Me_4_pyr)Li, [Mg{*µ*-C_4_H_2_N(2-CH_2_NH*t*Bu)}{N(SiMe_3_)_2_}]_2_, and (AlMe_3_)(*µ*-C_4_H_3_N)[2-CH_2_NH(*t*Bu)Li(tmeda)] [70,71,72,73].

**Figure 4:**
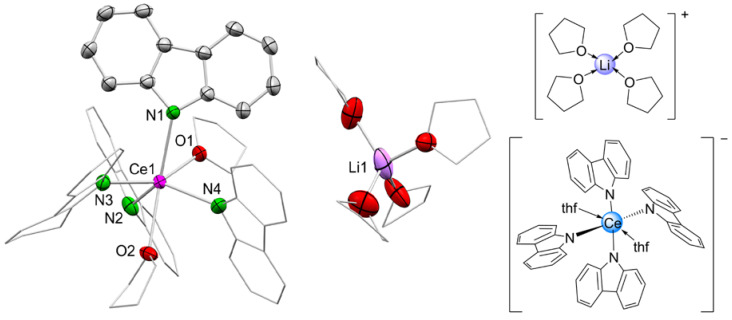
Crystal structure of [Ce(cbz)_4_(thf)_2_][Li(thf)_4_] (**5**). Ellipsoids are shown at the 50% probability level. Hydrogen atoms and disordered atoms are omitted for clarity. Selected interatomic distances (Å) and angles (°): Ce1−N1 2.531(4), Ce1−N2 2.480(4), Ce1−N3 2.497(4), Ce1−N4 2.484(4), Ce1−O1 2.577(4), Ce1−O2 2.583(3); N1−Ce1−N2 128.14(14), N1−Ce1−N3 90.91(13), N1−Ce1−N4 92.69(13), and O1−Ce1−O2 129.06(11).

Complex **5** was recrystallized from a 1:1 mixture of THF and Et_2_O to yield off-white crystals suitable for XRD analysis. The crystal structure of **5** shows a six-coordinate cerium center surrounded by four carbazolyl ligands, two THF donor molecules and a [Li(thf)_4_]^+^ counter ion (Figure 4). As seen before, the Ce1–N distances (2.480(4) to 2.531(4) Å) are slightly elongated compared with other separated ion-pair type ate complexes due to the higher coordination number of 6 compared with 4.

Targeted oxidations of the ate complexes **4** and **5** with *p*-benzoquinone led to an immediate color change to dark green and dark purple, respectively. Although indicative of a redox reaction, the isolation and characterization of a tetravalent cerium pyrrolate or carbazolate was unsuccessful. Dissolving the resulting crude product in THF-*d*_8_ resulted in the decolorization of the solution and the obtained ^1^H NMR spectrum showed only paramagnetically broadened and shifted signals. This play of colors clearly indicated a reduction in the putatively formed tetravalent species. The use of other solvents was not possible due to the insolubility of the cerium pyrrolates and carbazolates in nondonating solvents.

### 2.3. Reactivity toward CO_2_

**Figure 5:**
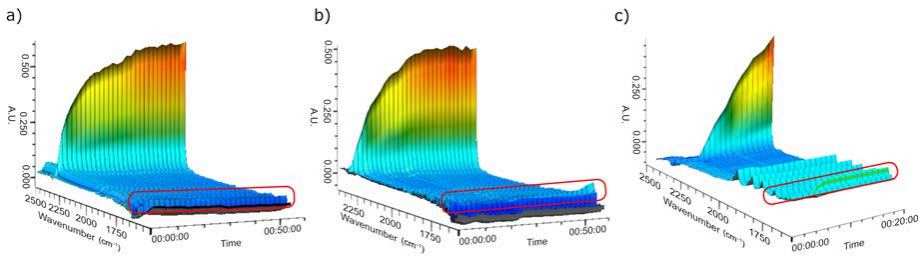
In situ IR spectroscopic measurements of the reaction of CO_2_ and (**a**) Ce(*t*Bu_2_pz)_4_ (**1**), (**b**) Ce(Ph_2_pz)_4_ (**2**), and (**c**) Ce(*t*BuMepz)_4_ (**3**) at −20 °C. The wavenumber of the C=O band is marked with a red box.

**Figure 6:**
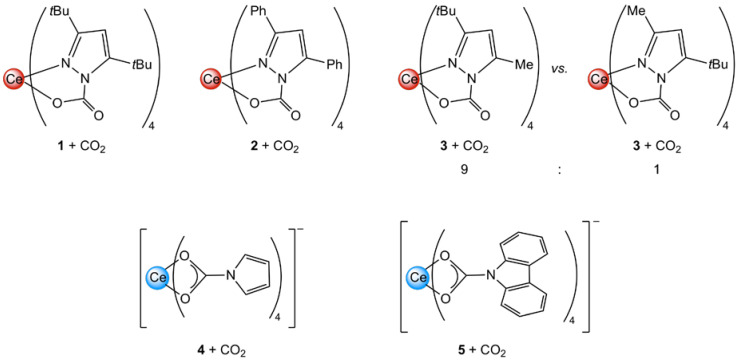
Putative insertion products of the reaction of complexes **1** to **5** with CO_2_.

Preliminary studies on the reactivity of the silylamide Ce[N(SiHMe_2_)_2_]_4_ (red color) [74] as well as Ce(N*i*Pr_2_)_4_ (purple) [75] and Ce(N*i*Pr_2_)_4_Li(thf) (orange) [75], bearing the very basic diisopropylamido ligand (Figure 1), with CO_2_ resulted in an instant decolorization of the solutions and the formation of colorless precipitates. ^1^H NMR spectroscopic measurements of the reaction mixtures provided inconclusive results. As noted in the Introduction, for rare-earth–metal silylamides, the formation of siloxide species and the elimination of isocyanates via silyl migration has been previously described [41,42,43]. Therefore, we focused on cerium pyrazolates and pyrrolates and their reactivity toward CO_2_. The possible insertion of CO_2_ into the Ce−N(pyrazolato) bond was examined via in situ IR spectroscopic measurements in toluene. Because compounds **1** and **2** did not display any insertion of CO_2_ at ambient temperature, the reactions were conducted at −20 °C. It was shown previously for dimethyl pyrazolate [Ce(Me_2_pz)_4_]_2_ that the CO_2_ insertion is more efficient at lower temperatures [38]. These experiments revealed no (**1**), slow (**2**) and fast insertion (**3**), as indicated by an increasing intensity of the characteristic C−O vibrations at around 1600 to 1750 cm^−1^ in the DRIFT (diffuse reflectance infrared Fourier transform) spectra (Figure 5). However, the insertion processes were slower and less efficient than observed previously for the dimethyl pyrazolate [Ce(Me_2_pz)_4_]_2_ [38]. ^1^H NMR spectroscopic investigations revealed only minor insertion of CO_2_ into the Ce−N(pyrazolato) bond of complex **1** (Figure S7, Supporting Information). Notwithstanding, a new signal set appeared assignable to a *t*Bu_2_pzCO_2_ ligand (Figure 6), displaying two singlets at 1.02 and 1.50 ppm for the *t*Bu-groups and one singlet at 5.97 ppm for the C−H proton of the pyrazole backbone. For the reaction of compound **2** with CO_2_, the respective ^1^H NMR spectrum showed a shift of all signals, indicating complete conversion (Figure S8, Supporting Information). However, unlike what was expected for an asymmetrically CO_2_-inserted pyrazolato ligand, a splitting of the phenyl signals was not detected. For the reaction of compound **3** with CO_2_, the formation of two signal sets with a ratio of 9:1 was observed (Figure S9, Supporting Information). Both sets displayed singlets for the *t*Bu and Me groups and one for the pyrazole C−H. This signal pattern might have resulted from a favorable insertion of CO_2_ into the methyl site of the pyrazolato ligand due to the lower steric hindrance (Figure 6). These results and previous findings clearly show that the steric demand of the substituents on the pyrazolato ligands is the key factor in the CO_2_-insertion reaction [38]. Therefore, the efficiency of the CO_2_ insertion with complexes [Ce(RR’pz)_4_] increases in the order of *t*Bu_2_pz < Ph_2_pz < *t*BuMepz < Me_2_pz. Unfortunately, any crystallization and structural elucidation of a CO_2_-inserted product was not successful.

**Figure 7:**
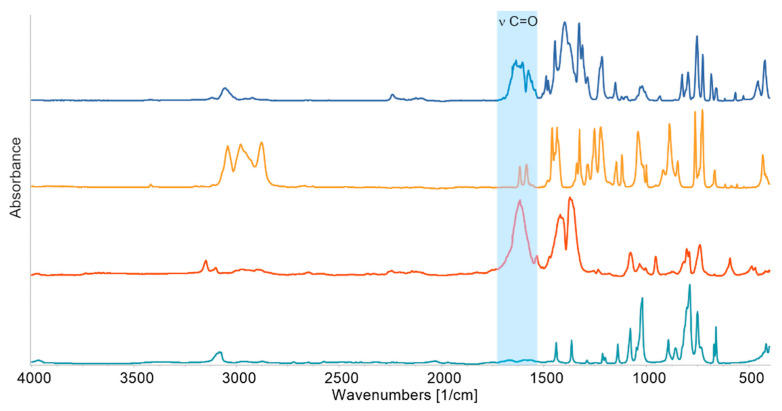
Stacked DRIFT (diffuse reflectance infrared Fourier transform) spectra of the reactions of **4** before (green trace) and after treatment with CO_2_ (orange trace) as well as **5** before (yellow) and after treatment with CO_2_ (blue) from bottom to top. The region for C−O vibrations is highlighted in blue.

Similarly, pyrrole-derived complexes **4** and **5** were treated with CO_2_ resulting in a shift of the signals in the ^1^H NMR spectra (Figures S10 and S11, Supporting Information). For pyrrolate **4**, two broadened singlets were observed at 6.82 and 9.99 ppm, indicating the formation of a carbamato-like ligand CO_2_∙pyr (Figure 6). Similar ligand formation was already found in the reaction of *n*BuLi, pyrrole, and carbon dioxide [76]. The ^1^H NMR spectrum of the reaction of carbazolate **5** with CO_2_ shows various paramagnetically broadened and shifted signals ruling out any conclusive interpretation. Nevertheless, the DRIFT spectra of the crude products, after removal of all volatiles under reduced pressure, clearly revealed an intense band for the C−O vibrations at 1600 to 1750 cm^−1^ (Figure 7). This finding also indicated that, unlike [Ce(Me_2_pz)_4_]_2_ [38], the CO_2_ insertion into the Ce−N(pyrrolato) bond is not reversible at ambient temperature under reduced pressure. Regrettably, a structural elucidation of the reaction products was not feasible.

### 2.4. Difference between Pyrazolates and Pyrrolates/Carbazolates

**Scheme 3:**
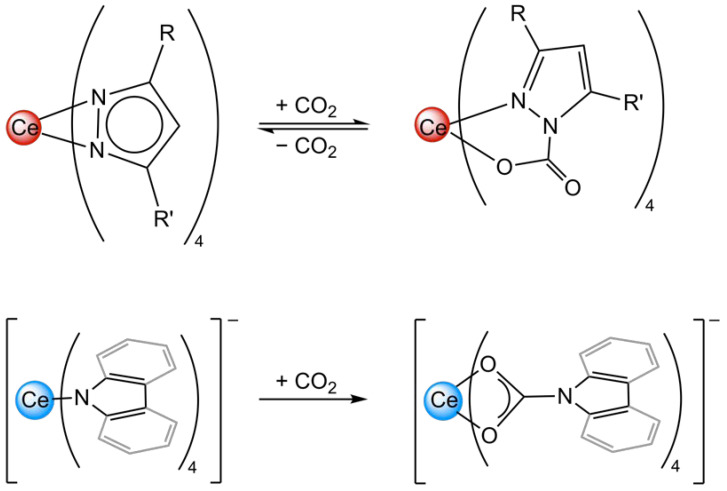
Reversible and irreversible CO_2_ insertion into Ce−N(pz) and Ce−N(pyr)/Ce−N(cbz) bonds, respectively.

Based on our findings, it can be hypothesized that CO_2_ insertion into the Ce−N(RR’pz) bond is mainly affected by the sterics of the pyrazole substituents. This is clearly indicated by the performance of [Ce(Me_2_pz)_4_]_2_ being far superior to that of Ce(*t*Bu_2_pz)_4_. Accordingly, any significant electronic effect caused by differently alkyl-substituted pyrazolato ligands can be ruled out. However, our results suggest that the reversibility of the CO_2_ insertion is most likely driven by the two adjacent *N*-donor atoms of the pyrazolato ligand, preserving a Ce–N coordination also in the Ce−pz∙CO_2_ fragments (Scheme 3). Apparently, the formation of a five-membered heterocyclic ring with *κ*^2^(*N*,*O*) coordination mode is outperforming a four-membered ring with terminal *κ*^2^(*O*,*O*) carboxylato coordination. The latter *κ*^2^(*O*,*O*) coordination would result from the insertion of CO_2_ into the Ce−N(pyr) or Ce−N(cbz) bonds (Scheme 3). Note that any reversible CO_2_ insertion has not yet been evidenced for such simple carbamates.

## 3. Materials and Methods

### 3.1. General Procedures

All manipulations were performed under an inert atmosphere (Ar) using a glovebox (MBraun 200B; <0.1 ppm O_2_, <0.1 ppm H_2_O), or according to standard Schlenk techniques in oven-dried glassware. The solvents were purified with Grubbs-type columns (MBraun SPS, solvent purification system) and stored in a glovebox. Ce[N(SiHMe_2_)_2_]_4_Li(thf), Ce[N(SiHMe_2_)_2_]_4_, and Ce(*t*Bu_2_pz)_4_ (**1**) were synthesized according to published procedures [65,66,67]. HMe_2_pz, H*t*Bu_2_pz, pyrrole, and carbazole were purchased from Sigma Aldrich (St. Louis, MO, USA) and used as received. HPh_2_pz and H*t*BuMepz were synthesized according to the literature [77]. C_6_D_6_, toluene-*d*_8_, and THF-*d*_8_ were purchased from Euriso-top (Saint-Aubin, France) and pre-dried over NaK alloy and filtered off prior use; THF-*d*_8_ was re-condensed. NMR spectra were recorded at 26 °C on a Bruker AVII+400 (^1^H: 400.13 MHz) or a Bruker AVIIIHD-300 (^1^H: 300.13 MHz, ^7^Li 116.64 MHz) using J. Young-valved NMR tubes. ^1^H NMR shifts are referenced to a solvent resonance and reported in parts per million (ppm) relative to tetramethylsilane. ^7^Li NMR spectra are reported relative to LiCl. Analyses of NMR spectra were performed with ACD/NMR Processor Academic Edition (product version: 12.01). Infrared spectra were recorded on a ThermoFisher Scientific (Waltham, MA, USA) NICOLET 6700 FTIR ($\tilde{\nu }$ = 4000–400 cm^−1^) spectrometer using a DRIFTS chamber with dry KBr/sample mixtures and KBr windows. Elemental analysis (C, H, and N) was performed on an Elementar vario MICRO cube. In situ IR spectra were recorded on a METTLER TOLEDO (Columbus, OH, USA) ReactIR 15. For details on XRD analyses and crystallographic data, see Supporting information.

### 3.2. Synthesis of Ce(Ph_2_pz)_4_∙tol (***2***)

Ce[N(SiHMe_2_)_2_]_4_ (0.40 g, 0.60 mmol) in *n*-hexane (2 mL) was added to a suspension of HPh_2_pz (0.33 g, 2.4 mmol) in toluene (2 mL). After 1 h, all volatiles were removed under reduced pressure. Crystallization from concentrated toluene solutions produced **2** as dark purple crystals. Yield: 0.52 g (0.47 mmol, 79%). ^1^H NMR (C_6_D_6_, 400.13 MHz, 26 °C): = 7.78 (m, 16 H, C−H Ph), 7.11 (s, 4 H, C−H pz), 6.91 (m, 24 H, C−H Ph) ppm; IR (DRIFT): $\tilde{\nu }$ = 3064 (w), 3036 (w), 2916 (w), 1944 (w), 1883 (w), 1807 (w), 1755 (w), 1682 (w), 1602 (w), 1562 (w), 1489 (w), 1467 (s), 1421 (m), 1401 (m), 1335 (w), 1282 (w), 1254 (w), 1151 (w), 1103 (w), 1072 (w), 1018 (m), 999 (m), 965 (m), 914 (w), 837 (w), 809 (w), 760 (s), 731 (s), 700 (s), 692 (m), 678 (m), 666 (w), 537 (w), 481 (w), 465 (w), 429 (m), 422 (m), 405 (w) cm^−1^; elemental analysis (%) calcd. for C_67_H_52_CeN_8_ (1109.33): C 72.54, H 4.73, N 10.10; found: C 72.40, H 4.52, N 10.68.

### 3.3. Synthesis of [Ce(tBuMepz)_4_]_n_ (***3***)

Ce[N(SiHMe_2_)_2_]_4_ (0.40 g, 0.60 mmol) in toluene (2 mL) was added to a suspension of H*t*BuMepz (0.53 g, 2.4 mmol) in toluene (2 mL). After 2 h, all volatiles were removed in vacuo, producing **3** as a dark red sticky solid. Yield of the crude product: 0.41 g. ^1^H NMR (C_6_D_6_, 400.13 MHz, 26 °C): = 6.15 (s, 4 H, C−H pz), 2.22 (s, 12 H, Me), 1.24 (s, 36 H, *t*Bu) ppm; IR (DRIFT): $\tilde{\nu }$ = 3105 (w), 2961 (vs), 2924 (s), 2901 (s), 2862 (m), 1558 (w), 1508 (s), 1474 (m), 1458 (m), 1425 (s), 1387 (m), 1362 (m), 1291 (w), 1237 (s), 1210 (w), 1124 (w), 1084 (w), 1032 (w), 1020 (w), 994 (m), 962 (w), 819 (w), 797 (m), 718 (w), 700 (w), 686 (w), 567 (w), 507 (m) cm^−1^; elemental analysis (%) calcd. for C_32_H_52_CeN_8_ (688.94): C 55.79, H 7.61, N 16.26; found: C 57.17, H 7.49, N 16.29. The slightly increased carbon value might be a result of retained solvent.

### 3.4. Synthesis of [Ce_2_(pyr)_6_(µ-pyr)_2_(thf)_2_][Li(thf)_4_]_2_ (***4***)

Pyrrole (141 mg, 2.11 mmol) in *n*-hexane (3 mL) was added to a stirred solution of Ce[N(SiHMe_2_)_2_]_4_Li(thf) (394 mg, 0.526 mmol) in *n*-hexane (5 mL). Immediately, a colorless precipitate was formed. After 1 h, all volatiles were removed under reduced pressure. The resulting solid was recrystallized from THF, producing **4** as colorless crystals. Yield: 189 mg (0.122 mmol, 46%). ^1^H NMR (THF-*d_8_*; 400.11 MHz, 26 °C) δ = 7.39 (bs), 4.23 (bs) ppm; ^7^Li NMR (THF-*d_8_*, 116.64 MHz, 25 °C) δ = 2.0 ppm. DRIFTS: $\tilde{\nu }$ = 3085 (w), 1441 (w), 1365 (w), 1213 (vw), 1200 (vw), 1140 (w), 1078 (m), 1048 (w), 1021 (s), 894 (w), 858 (w), 789 (vs), 751 (m), 670 (w), 660 (m), 416 (w) cm^−1^; elemental analysis (%) calcd. for C_72_H_112_Ce_2_Li_2_N_8_O_10_ (1543.85): C 56.02, H 7.31, N 7.36; found: C 46.96, H 3.90, N 13.74. Calculated without THF donor ligands for C_42_H_54_Ce_2_N_8_O_2_ (983.18): C 51.31, H 5.54, N 11.40. On multiple attempts, no better elemental analysis could be obtained.

### 3.5. Synthesis of [Ce(cbz)_4_(thf)_2_][Li(thf)_4_] (***5***)

Ce[N(SiHMe_2_)_2_]_4_Li(thf) (406 mg, 0.557 mmol) in *n*-hexane (5 mL) was added to a stirred suspension of carbazole (363 mg, 2.23 mmol) in *n*-hexane (5 mL). After 1 h, a yellow precipitate formed, and volatiles were removed under reduced pressure. The resulting solid was recrystallized from THF/Et_2_O (1:1), giving **5** as off-white crystals. Yield: 486 mg (0.415 mmol, 74%). ^1^H NMR (THF-*d_8_*; 400.11 MHz, 26 °C) δ = 8.64 (d), 7.90 (bs), 7.70 (d), 7.32 (d), 7.02 (bs), 6.70 (bs) 6.28 (s), 6.04 (s), 5.30 (s), 4.22 (d), 3.62 (s, α-CH(thf)), 1.77 (s, β-CH(thf)), 0.33 (bs), −10.16 (bs) ppm; ^7^Li NMR (THF-*d_8_*, 116.64 MHz, 25 °C) δ: −0.3 ppm. DRIFTS: $\tilde{\nu }$ = 3418 (vw), 3054 (w), 2979 (w), 2878 (w), 1635 (m), 1576 (m), 1541 (m), 1489 (m), 1479 (m), 1447 (s), 1395 (vs), 1329 (vs), 1312 (s), 1287 (m), 1216 (m), 1151 (w), 1121 (vw), 1043 (w), 934 (vw), 889 (vw), 826 (m), 797 (m), 753 (s), 725 (m), 684 (m), 658 (w), 617 (vw), 567 (vw), 526 (vw), 458 (w), 425 (m) cm^−1^; elemental analysis (%) calcd. for C_72_H_80_CeLiN_4_O_6_ (1244.51): C 69.49, H 6.48, N 4.50; found: C 68.42, H 6.42, N 3.97.

## 4. Conclusions

The feasibility of CO_2_ insertion reactions was further substantiated for homoleptic ceric pyrazolates [Ce(RR’pz)_4_]. Crucially, with increasing bulkiness of the substituents R/R’ on the pyrazolato ligand, the insertion of carbon dioxide becomes increasingly hindered; therefore, the least bulky dimethyl-substituted pyrazolato ligand performed best in CO_2_ insertions. En route to homoleptic Ce^IV^ pyrrolates and carbazolates, we were able to isolate cerous ate complexes [Ce_2_(pyr)_6_(*µ*-pyr)_2_(thf)_2_][Li(thf)_4_]_2_ and [Ce(cbz)_4_(thf)_2_][Li(thf)_4_]. Such Ce(III) pyrrolates and carbazolates are also capable of CO_2_ activation, but most likely in an irreversible manner. Oxidation attempts to form ceric pyrrolate or carbazolate compounds were conducted; however, the structural elucidation of any Ce^IV^ species was not feasible.

## Data Availability

Not applicable.

## Supplementary Materials

The following are available online. The ^1^H (**2**, **3**, **4**, and **5,** and their reactions with CO_2_) and ^7^Li (**4** and **5**). NMR spectra as well as selected crystallographic data for compounds **2**, **4**, and **5** are available online. Complete crystallographic data can be obtained free of charge from The Cambridge Crystallographic Data Centre via www.ccdc.cam.ac.uk/data_request/cif, Deposition Number 2069959-2069961.

## Sample Availability

Samples of the compounds are not available from authors.
