# Supplementary material for: Effect of Substituents of Cerium Pyrazolates and Pyrrolates on Carbon Dioxide Activation
Source: Molecules. 2021 Mar 31;26(7):1957. doi: 10.3390/molecules26071957 (PMC8037029; doi:10.3390/molecules26071957)
Supplement: Supplementary file 1 [file molecules-26-01957-s001.pdf]

molecules

MDPI

Supplementary Information

Effect of Substituents of Cerium Pyrazolates on Carbon Dioxide Activation

Uwe Bayer, Adrian Jenner, Jonas Riedmaier, Cäcilia Maichle-Mössmer, and Reiner Anwander*

1) *NMR Spectra (solvent signals are marked with *)*

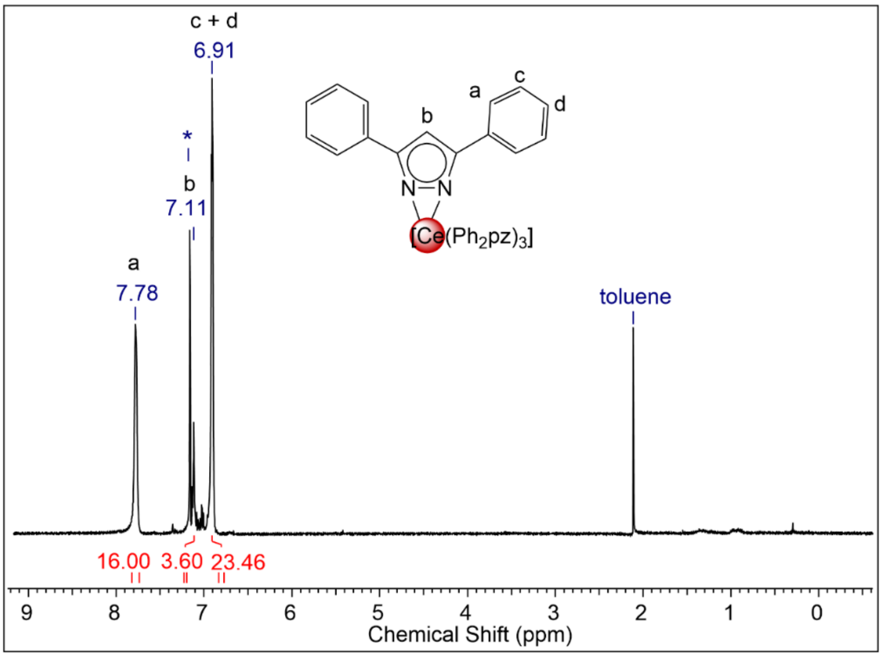

Figure S1. ${}^{1}H$ NMR spectrum (C_6D_6, 400.13 MHz, 26 °C) of **2**.

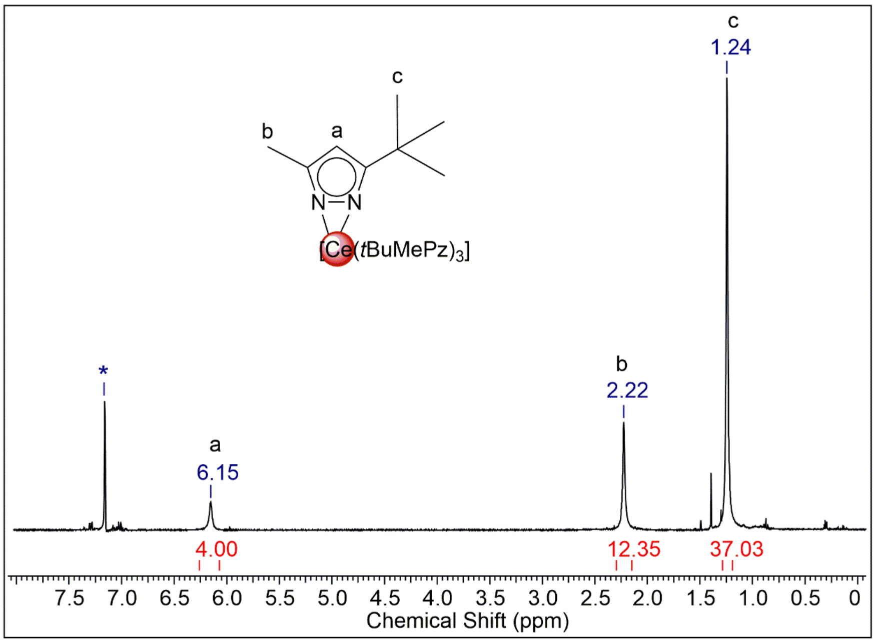

Figure S2. ${}^{1}H$ NMR spectrum (C_6D_6, 400.13 MHz, 26 °C) of **3**.

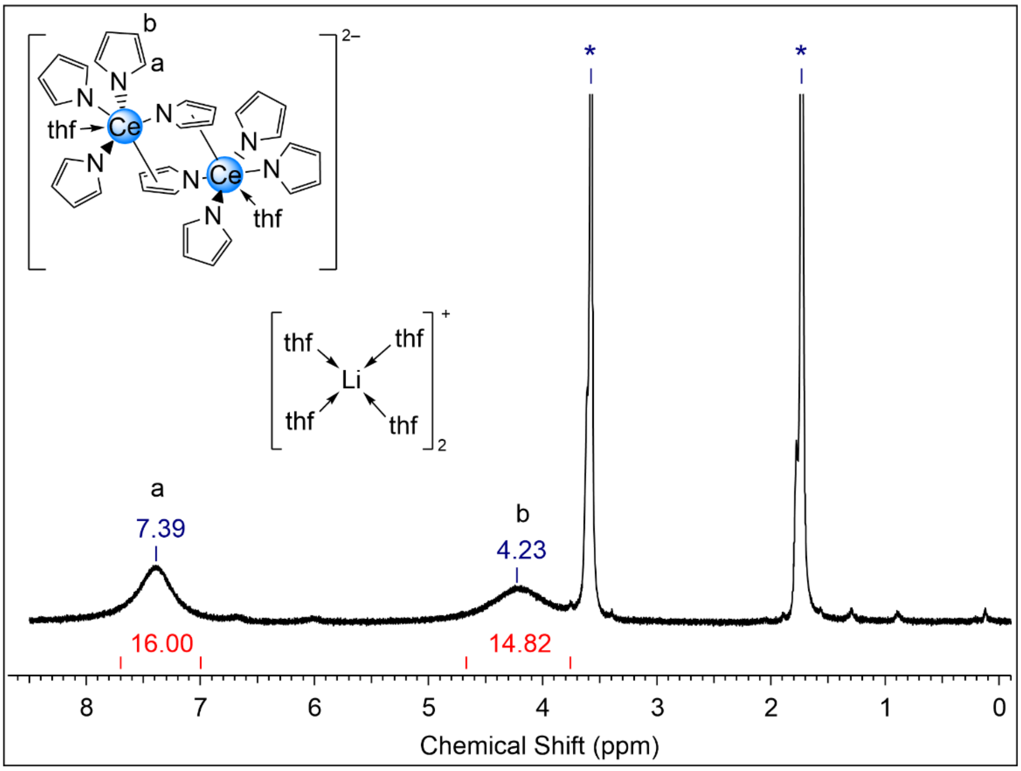

Figure S3. ^{1}H NMR spectrum (THF-d_8, 400.13 MHz, 26 °C) of **4**.

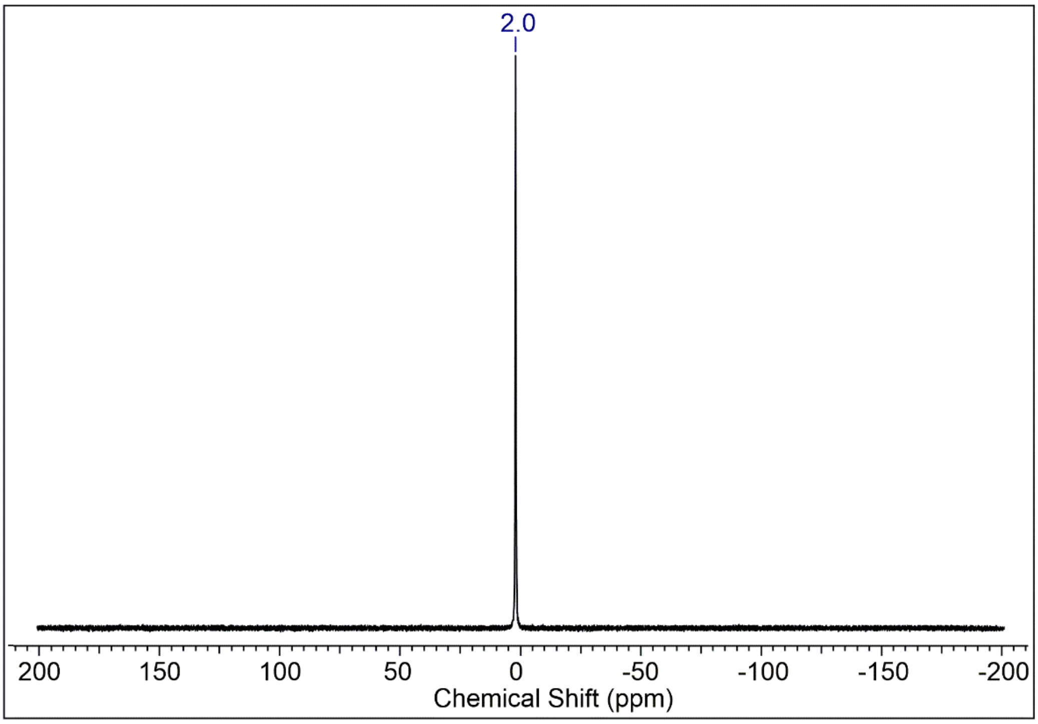

Figure S4. ^{7}Li NMR spectrum (THF-d_8, 116.64 MHz, 26 °C) of **4**.

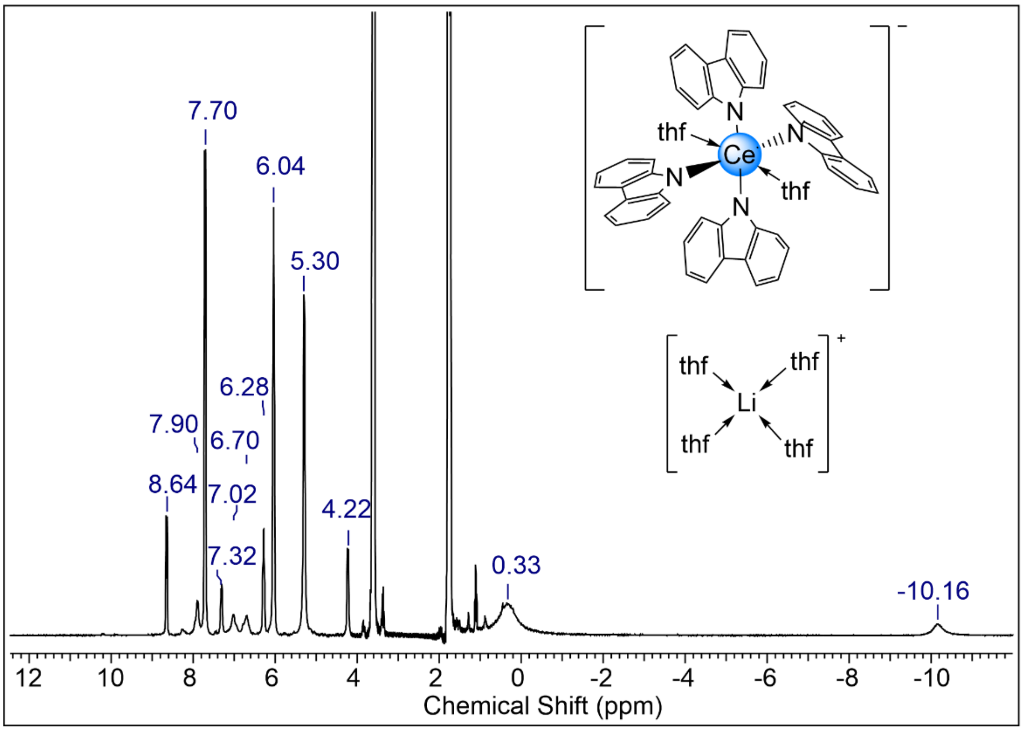

Figure S5. ^{1}H NMR spectrum (THF-d_8, 400.13 MHz, 26 °C) of **5**.

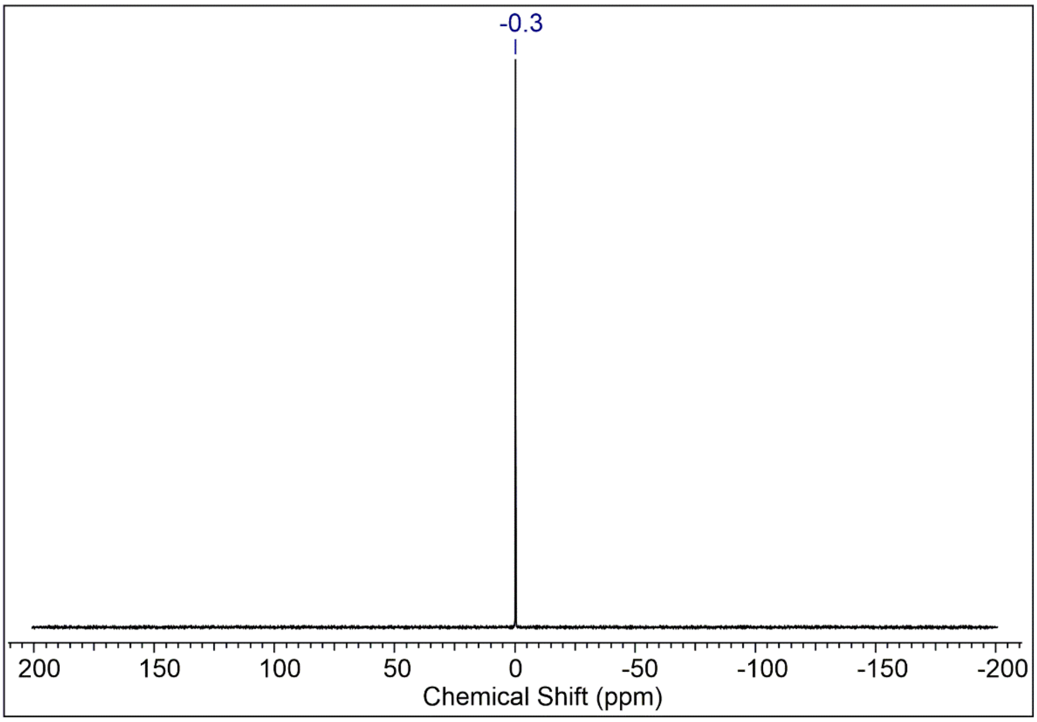

Figure S6. ^{7}Li NMR spectrum (THF-d_8, 116.64 MHz, 26 °C) of **5**.

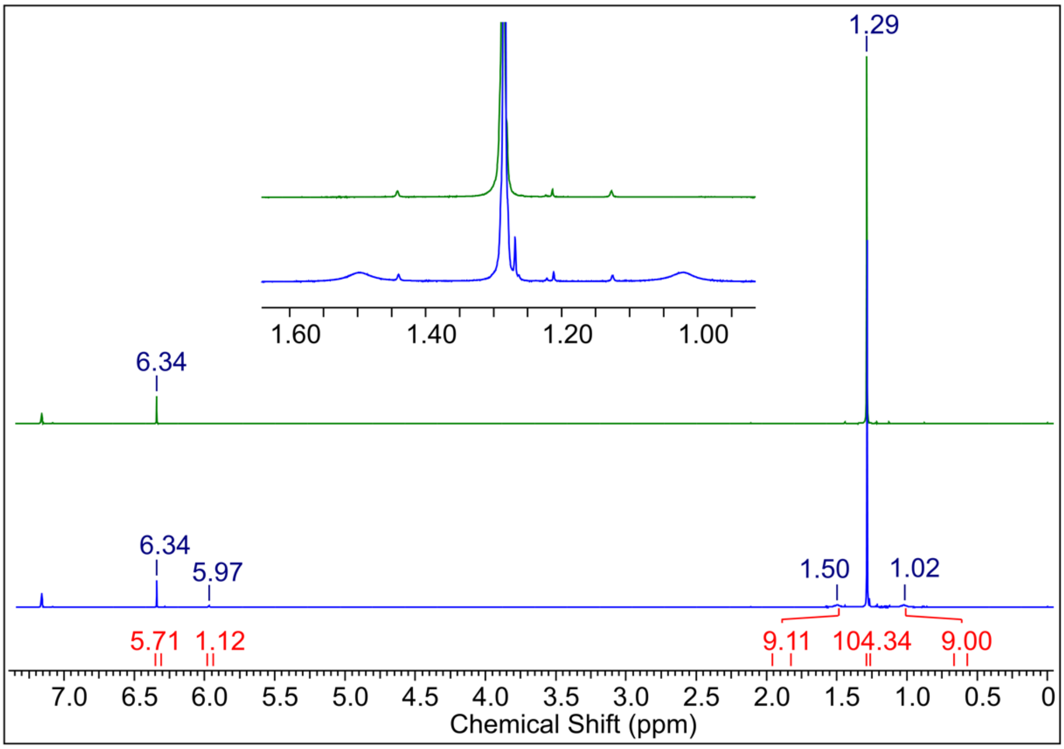

Figure S7. Stacked ^{1}H NMR spectra (C_6D_6, 400.13 MHz, 26 °C) of **1** (green trace, top) and **1** + CO_2 (blue trace, bottom).

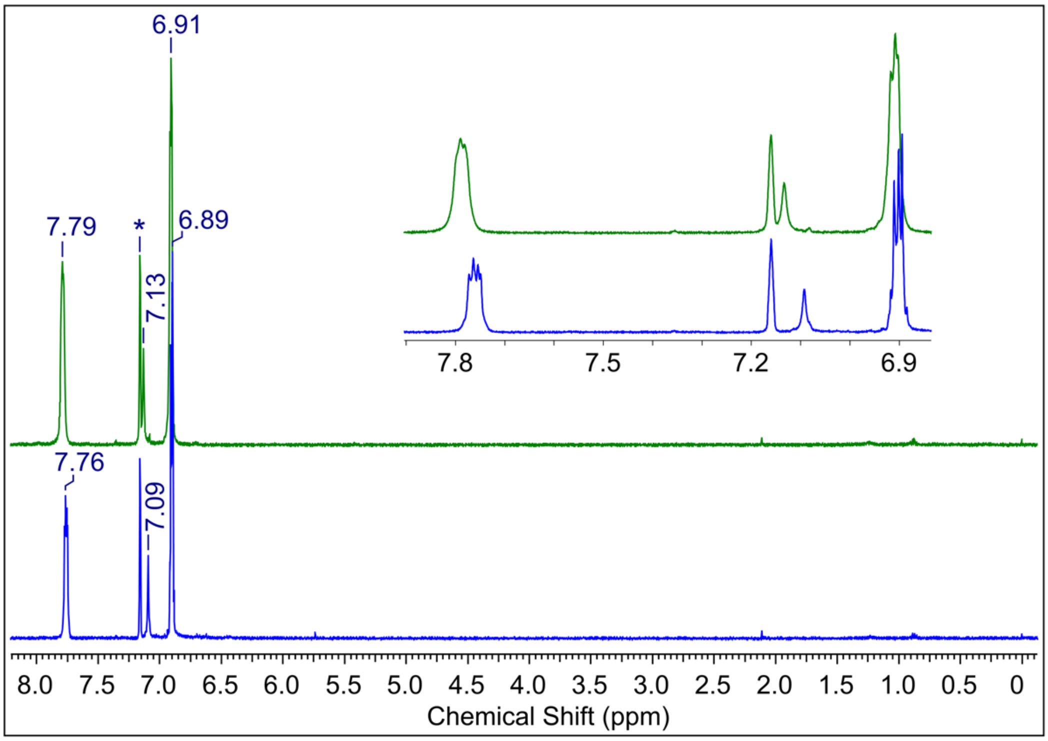

Figure S8. Stacked ^{1}H NMR spectra (C_6D_6, 400.13 MHz, 26 °C) of **2** (green trace, top) and **2** + CO_2 (blue trace, bottom).

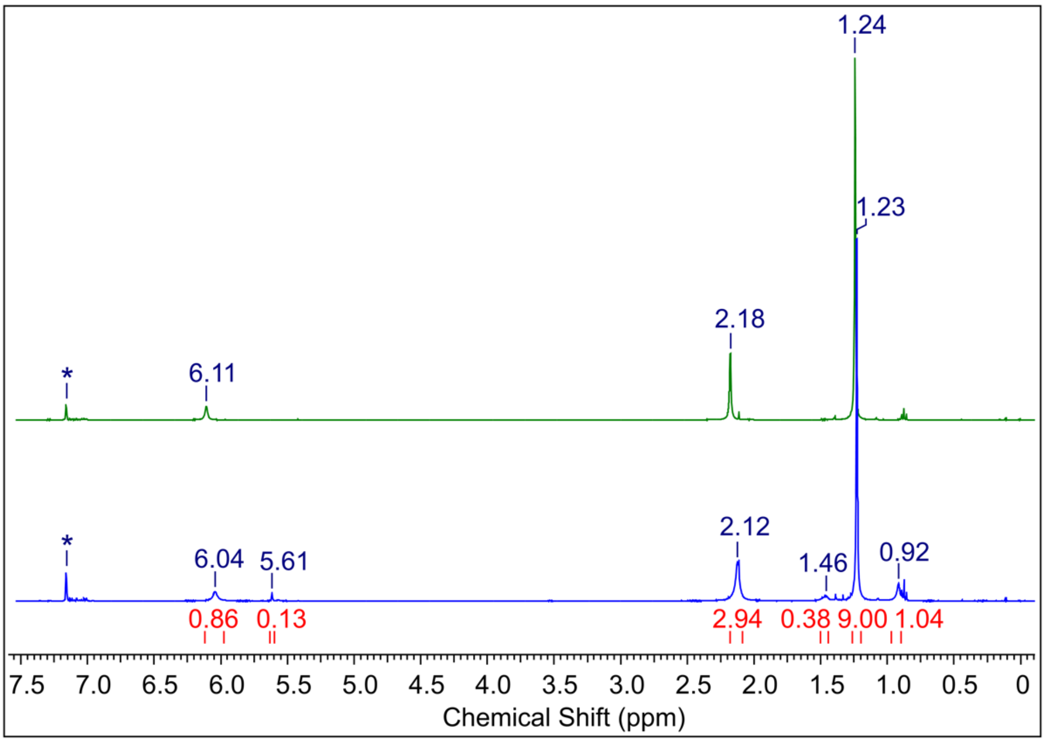

Figure S9. Stacked ^{1}H NMR spectra (C_6D_6, 400.13 MHz, 26 °C) of **3** (green trace, top) and **3** + CO_2 (blue trace, bottom).

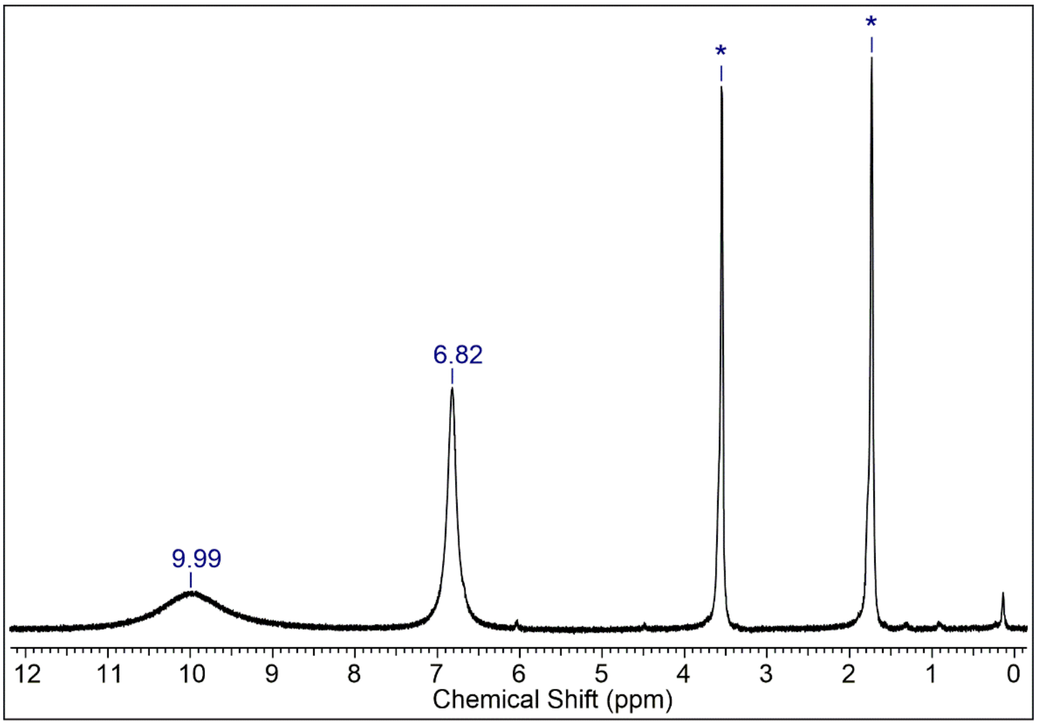

Figure S10. ^{1}H NMR spectrum (THF-*d*8, 400.13 MHz, 26 °C) of **4** + CO_2.

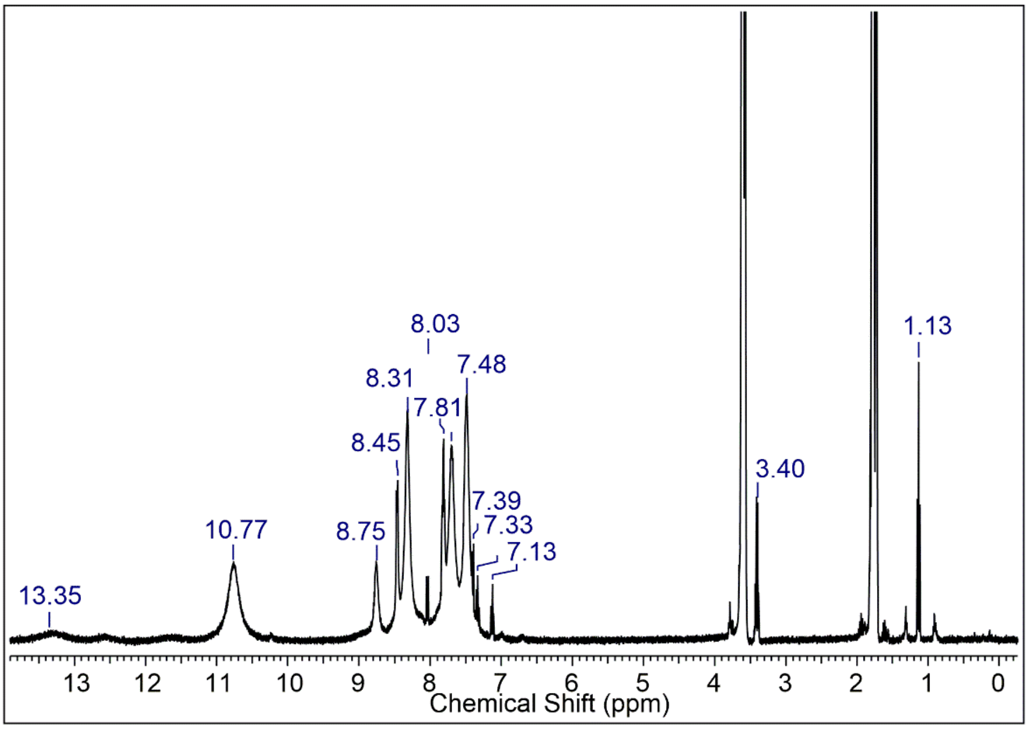

Figure S11. ^{1}H NMR spectrum (THF-*d*$_8$, 400.13 MHz, 26 °C) of **5** + CO_2.

2) *Crystallographic Data*

Crystals for X-ray structure analysis were grown using saturated solutions of toluene/*n*-hexane (**2**), THF/Et_2O (**4**, **5**). Suitable crystals for were handpicked in a glovebox, coated with Parabar 10312 and stored on microscope slides, and mounted rapidly outside the glovebox onto a micro loop. Data collection was done on a Bruker APEX II Duo diffractometer by using QUAZAR optics and Mo Kα (λ = 0.71073 Å). The data collection strategy was determined using COSMO [1] employing ω scans. Raw data were processed by APEX 3 [2] and SAINT [3], corrections for absorption effects were applied using SADABS [4]. The structures were solved by direct methods and refined against all data by full-matrix least-squares methods on F^2 using SHELXTL [5] and SHELXLE [6]. All atoms were refined anisotropically. Disorder models are calculated using DSR [7], a program included in ShelXle, for refining disorder.

Plots were generated by using CCDC Mercury 3.19.1 [8]. Further details regarding the refinement and crystallographic data are listed in Table S1 and in the CIF files.

Table S1. Crystallographic data for compounds **2**, **4**, and **5**

	**2**	**4**	**5**
**formula**	$C_{74}H_{60}CeN_8$	$C_{72}H_{112}Ce_2Li_2N_8O_{10}$	$C_{72}H_{80}CeLiN_4O_6$
**CCDC**	2069960	2069959	2069961
**M [g·mol⁻¹]**	1201.42	1543.81	1244.46
**λ [Å]**	0.71073	0.71073	0.71073
**color**	purple	colorless	colorless
**crystal dimensions [mm]**	0.500 x 0.095 x 0.092	0.172 x 0.150 x 0.113	0.259 x 0.153 x 0.080
**crystal system**	orthorhombic	triclinic	triclinic
**space group**	Pbcn	$P\bar{1}$	$P\bar{1}$
**a [Å]**	23.7563(12)	11.6442(14)	12.3890(10)
**b [Å]**	12.7848(7)	13.6044(16)	13.5313(11)
**c [Å]**	19.3528(10)	14.1370(17)	18.6323(15)
**α [°]**	90	61.905(3)	88.927(2)
**β [°]**	90	71.159(3)	88.581(2)
**γ [°]**	90	86.648(3)	81.026(2)
**V [Å³]**	5877.8(5)	1857.9(4)	3084.0(4)
**Z**	4	1	2
**F(000)**	2472	802	1298
**T [K]**	173(2)	100(2)	100(2)
**ϱ_{calcd} [g·cm⁻³]**	1.358	1.380	1.340
**μ[mm⁻¹]**	0.827	1.269	0.795
**Data / restraints / parameters**	7918 / 123 / 442	10020 / 0 / 424	14249 / 1585 / 997
**Goodness of fit**	1.017	1.022	1.041
**R_1 (I > 2σ (I))[a]**	0.0297	0.0353	0.0657
**$_{\omega}R_2$ (all data)[b]**	0.0811	0.0823	0.1804

[a] $R_1 = \Sigma(||F0|-|Fc||)/\Sigma|F0|$,F0> 4s(F0). $_{\omega}R_2 = \{\Sigma[w(F02-Fc2)2/\Sigma[w(F02)^2]\}^{1/2}$

3) *References*

1. *COSMO, v. 1.61*; Bruker AXS Inc.: Madiso, Wi, 2012.
2. *APEX 3*, 2017.3-0, Bruker AXS Inc., Madison, WI, 2017.
3. *SAINT v. 8.38A*, Bruker AXS Inc., Madison, WI, 2017.
4. *SADABS* Krause, L., Herbst-Irmer, R., Sheldrick, G. M. & Stalke, D. *J. Appl. Cryst.* **2015**, *48*, 3-10.
5. Sheldrick G.M., *SHELXT Acta Cryst.* **2015**, *A71*, 3-8.
6. Hübschle, C.B.; Sheldrick, G.M.; Dittrich, B. *ShelXle*: A Qt Graphical User Interface for *SHELXL*. *J. Appl. Crystallogr.* **2011**, *44*, 1281–1284, doi:10.1107/S0021889811043202.
7. Kratzert, D.; Holstein, J.J.; Krossing, I. *DSR*: Enhanced Modelling and Refinement of Disordered Structures with *SHELXL*. *J. Appl. Crystallogr.* **2015**, *48*, 933–938, doi:10.1107/S1600576715005580.
8. Macrae, C.F.; Bruno, I.J.; Chisholm, J.A.; Edgington, P.R.; McCabe, P.; Pidcock, E.; Rodriguez-Monge, L.; Taylor, R.; van de Streek, J.; Wood, P.A. *Mercury CSD 2.0* – New Features for the Visualization and Investigation of Crystal Structures. *J. Appl. Crystallogr.* **2008**, *41*, 466–470, doi:10.1107/S0021889807067908.
